# Supplementary material for: Content-rich biological network constructed by mining PubMed abstracts
Source: BMC Bioinformatics. 2004 Oct 8;5:147. doi: 10.1186/1471-2105-5-147 (PMC528731; doi:10.1186/1471-2105-5-147)
Supplement: Additional File 2 — The original results of the above study (non-essential files are deleted to keep the file size under the limit set by BMC bioinformatics). [file 1471-2105-5-147-S2.bz2 › chilibotAdditionalFile2/dip30/562ID11502794E10923/html/IGF2_IGF2R.html]

 


 **IGF2** and **IGF2R** 
  
Found 192 abstracts in PubMed, retrieved 30.  
 

 What does Google say? 
 PDF only 
| .edu only 

---

**Interactive relationship** (e.g. stimulation, inhibition, etc)

**Neutral relationship**- LNCaP cells expressing a mutant  **IGF2R**  incompetent to bind IGF II  [ **IGF2** ]  IGF2RMyc I T proliferated more rapidly than both vector transfected cells and cells expressing the IGF2RMyc.  Ref: 12586773 Endocrinology, 2003
- The IGF II  [ **IGF2** ]  mannose 6 phosphate Man 6 P receptor  **IGF2R**  binds IGF II  [ **IGF2** ]  and Man 6 P bearing ligands at distinct binding sites.  Ref: 12586773 Endocrinology, 2003
- The subcellular distribution and ability to internalize cell surface IGF II  [ **IGF2** ]  of IGF2RMyc were indistinguishable from endogenous  **IGF2R**  in PC 3 cells.  Ref: 12586773 Endocrinology, 2003
- In contrast, the IGF II  [ **IGF2** ]  cation independent mannose 6 phosphate receptor  [ **IGF2R** ]  regulates IGF II  [ **IGF2** ]  clearance.  Ref: 11739335 Endocr Rev, 2001
- We can, therefore, conclude that residues at positions 54 and 55 in IGF II  [ **IGF2** ]  are important for and equally contribute to  **IGF2R**  binding.  Ref: 11811790 Growth Factors, 2001
- The ability of the insulin receptor to act as a bona fide IGF II  [ **IGF2** ]  dependent growth promoter is underscored by its rescue of double knockout Igf1r  **Igf2r**  mice.  Ref: 11739335 Endocr Rev, 2001
- There is a 4 and 3.3 fold difference in dissociation constants for A54R IGF II  [ **IGF2** ]  and L55R IGF II  [ **IGF2** ] , respectively, and a 6.6 fold difference for A54R L55R IGF II  [ **IGF2** ]  compared with IGF II  [ **IGF2** ]  as measured by BlAcore analysis using purified rat  **IGF2R** .  Ref: 11811790 Growth Factors, 2001
- The underlying specificity of the interaction between insulin like growth factor II  [ **IGF2** ]  and mammalian Type 2 insulin like growth factor cation independent mannose 6 phosphate receptor  [ **IGF2R** ]  is not understood.  Ref: 11811790 Growth Factors, 2001
- **IGF2R**  contains 15 homologous extracellular domains, with domain 11 primarily responsible for IGF II  [ **IGF2** ]  binding.  Ref: 11867533 EMBO J, 2002
- Structural analysis identifies the putative IGF II  [ **IGF2** ]  binding site at one end of the beta barrel whilst crystal lattice contacts suggest a model for the full length  **IGF2R**  extracellular region.  Ref: 11867533 EMBO J, 2002
- The mannose 6 phosphate IGF II  [ **IGF2** ]  receptor MPR300  [ **IGF2R** ]  mediates sorting of lysosomal enzymes from the trans Golgi network to endosomes and endocytosis of hormones, for example, of IGF II  [ **IGF2** ] .  Ref: 11792812 J Cell Sci, 2001

**Non-interactive relationship** (e.g. studied together, co-existance, homology, etc.)

- These data suggest that the IGF II  [ **IGF2** ]  and Man 6 P binding functions of the  **IGF2R**  have opposing activities, with respect to growth of prostate cancer cells.  Ref: 12586773 Endocrinology, 2003
- No significant differences in IGF I, IGF II  [ **IGF2** ] , IGF binding protein IGFBP 1, IGFBP 3, soluble IGF II  [ **IGF2** ]  receptor levels  **IGF2R** , IGF II  [ **IGF2** ]   **IGF2R**  ratio, and relative amounts of IGFBP 3 circulating forms were found between CG and NCG children.  Ref: 11756646 Pediatr Res, 2002
- Mu 1A deficiency induces a profound increase in MPR300  [ **IGF2R** ]  IGF II  [ **IGF2** ]  receptor internalization rate.  Ref: 11792812 J Cell Sci, 2001
- Insulin like growth factor II  [ **IGF2** ]  receptor  **IGF2R**  is a multifunctional cell surface receptor implicated in tumour suppression.  Ref: 11867533 EMBO J, 2002
- Since the mannose 6 phosphate insulin like growth factor II  [ **IGF2** ]  receptor M6P  **IGF2R**  plays a central role in the intracellular transport and endocytosis of M6P containing lysosomal enzymes, a deficiency in functional M6P  **IGF2R**  may underlie the hypersecretion of procathepsin D in invasive tumors.  Ref: 12165733 Med Sci Monit, 2002
- We showed reliable and reproducible results in distinguishing overall methylation profiles of DMRs regions of human SNRPN, H19, MEST PEG1, LIT1,  **IGF2** , TSSC5, WT1 antisense, and mouse H19, Mest Peg1,  **Igf2R**  imprinted genes.  Ref: 12613258 Biotechniques, 2003
- Moreover, analysis of imprinted gene expression in a methyltransferase knockout Dnmt1 mouse has shown that the imprint is lost in a number of cases, resulting in either two silent alleles  **Igf2** ,  **Igf2r**  and Kvlqt or two expressed alleles H19, p57kip2, Snrpn, and Xist 0 12.  Ref: 12843456 Methods Mol Biol, 2001
- At  **Igf2r**  encoding insulin like growth factor 2  [ **IGF2** ]  receptor, a sequence called region 2 is needed for methylation of the active maternal allele.  Ref: 12740578 Nat Genet< MedlineTA>Nat Genet, 2001
- To determine when in germ cell development this biallelic expression phenomenon commences, we isolated migrating PGCs by flow cytometry and determined the allele specific expression of four imprinted genes Snrpn,  **Igf2** , H19 and  **Igf2r** .  Ref: 12049782 Mech Dev, 2002
- Familial aggregation of abnormal methylation of parental alleles at the  **IGF2**  H19 and  **IGF2R**  differentially methylated regions.  Ref: 12812984 Hum Mol Genet< MedlineTA>Hum Mol Genet, 2002
- We examined allelic methylation ratios at DMRs within the  **IGF2**  H19 and  **IGF2R**  loci in a panel of 48 three generation families.  Ref: 12812984 Hum Mol Genet< MedlineTA>Hum Mol Genet, 2002
- BACKGROUND Loss or mutation of the mannose 6 phosphate insulin like growth factor II  [ **IGF2** ]  receptor M6P  **IGF2R**  has been found in breast cancer.  Ref: 12149131 BMC Cancer, 2002
- The  **IGF2**  receptor  **IGF2R**  is a USF2 specific target in nontumorigenic mammary epithelial cells, but not in breast cancer cells.  Ref: 12857727 J Biol ChemJ Biol Chem, 2002
- Our purpose was to evaluate inherited short tandem repeat polymorphisms of the insulin like growth factor II  [ **IGF2** ]  receptor gene  **IGF2R**  in oral cancer risk.  Ref: 12543777 Cancer Res, 2003
- In addition, we have extended physical maps of sheep and goat as 11 genes HSD3B1, INHBA, CSN10,  **IGF2R** , PIGR, MAP1B, DSC1, ELN, TNFRSF6, CGN1,  **IGF2**  and 14 genes SOD1, HSD3B1, CSN10,  **IGF2R** , RB1, TG, PIGR, MAP1B, IGH@, LTF, DSC1, TNFRSF6, CGN1,  **IGF2**  were assigned for the first time to goat and sheep chromosomes, respectively.  Ref: 12873218 Anim Genet, 2003
- We also examined the methylation status of the imprinted genes  **Igf2r** , p57Kip2, Lit1, H19 and  **Igf2** .  Ref: 11776474 Differentiation, 2001
- We have mutated residues A54 and L55 of IGF II  [ **IGF2** ]  in the second A domain helix to arginine and measured  **IGF2R**  binding.  Ref: 11811790 Growth Factors, 2001
- Previous studies screening for quantitative trait loci QTLs that influence general intelligence in healthy individuals have identified four loci, two of which are located within the genes insulin like growth factor 2  [ **IGF2** ]  receptor  **IGF2R**  and the Msx1 homeobox.  Ref: 12556904 Mol Psychiatry, 2003
- Furthermore, decreased expression of M6P  **IGF2R**  enhanced IGF II  [ **IGF2** ]  induced proliferation and reduced cell susceptibility to TNF induced apoptosis.  Ref: 12149131 BMC Cancer, 2002
